# Natural history and comorbidities of generalised and partial lipodystrophy syndromes in Spain

**DOI:** 10.3389/fendo.2023.1250203

**Published:** 2023-11-16

**Authors:** Antía Fernández-Pombo, Sofía Sánchez-Iglesias, Ana I. Castro-Pais, Maria José Ginzo-Villamayor, Silvia Cobelo-Gómez, Teresa Prado-Moraña, Everardo Josué Díaz-López, Felipe F. Casanueva, Lourdes Loidi, David Araújo-Vilar

**Affiliations:** ^1^ Department of Psychiatry, Radiology, Public Health, Nursing and Medicine, IDIS-CiMUS, University of Santiago de Compostela, Santiago de Compostela, Spain; ^2^ Division of Endocrinology and Nutrition, University Clinical Hospital of Santiago de Compostela, Santiago de Compostela, Spain; ^3^ CIBER Fisiopatología de la Obesidad y la Nutrición (CIBERobn), Madrid, Spain; ^4^ Department of Estatística, Análise Matemática e Optimización, University of Santiago de Compostela, Santiago de Compostela, Spain; ^5^ Galician Public Foundation for Genomic Medicine (SERGAS-Xunta de Galicia), Santiago de Compostela, Spain

**Keywords:** generalised lipodystrophy, partial lipodystrophy, body composition, diabetes mellitus, hypertriglyceridaemia, atherosclerotic cardiovascular disease, mortality

## Abstract

The rarity of lipodystrophies implies that they are not well-known, leading to delays in diagnosis/misdiagnosis. The aim of this study was to assess the natural course and comorbidities of generalised and partial lipodystrophy in Spain to contribute to their understanding. Thus, a total of 140 patients were evaluated (77.1% with partial lipodystrophy and 22.9% with generalised lipodystrophy). Clinical data were collected in a longitudinal setting with a median follow-up of 4.7 (0.5-17.6) years. Anthropometry and body composition studies were carried out and analytical parameters were also recorded. The estimated prevalence of all lipodystrophies in Spain, excluding Köbberling syndrome, was 2.78 cases/million. The onset of phenotype occurred during childhood in generalised lipodystrophy and during adolescence-adulthood in partial lipodystrophy, with the delay in diagnosis being considerable for both cohorts. There are specific clinical findings that should be highlighted as useful features to take into account when making the differential diagnosis of these disorders. Patients with generalised lipodystrophy were found to develop their first metabolic abnormalities sooner and a different lipid profile has also been observed. Mean time to death was 83.8 ± 2.5 years, being shorter among patients with generalised lipodystrophy. These results provide an initial point of comparison for ongoing prospective studies such as the ECLip Registry study.

## 1 Introduction

Lipodystrophy syndromes are a heterogeneous group of rare disorders distinguished by the selective loss of adipose tissue. They may be generalised if the loss of adipose tissue affects the whole body, or partial if only part of the body is affected (1, 2). These generalised and partial lipodystrophy syndromes are also characterised by the ectopic accumulation of adipose tissue and the presence of insulin resistance and, consequently, by a variable degree of metabolic dysfunction, with diabetes mellitus (DM), dyslipidaemia, fatty liver disease, cardiovascular disease and reproductive dysfunction.

In 2017, the range of worldwide prevalence of all lipodystrophies (excluding human immunodeficiency virus (HIV)-related lipodystrophy) as determined by electronic medical record (EMR) database searches has been estimated at 1.3 to 4.7 cases per million inhabitants. However, it has also been estimated that for partial Mendelian lipodystrophies the estimated prevalence is higher (3, 4). The extreme rarity of lipodystrophy syndromes implies that they are not well-known. In addition, the clinical characterisation of these disorders is often deficient, there is a certain degree of phenotypical variability between the different subtypes of lipodystrophy and their aetiology is diverse, as are the pathogenetic mechanisms which lead to the alteration of adipose tissue. Thus, to date, they are considered to be underdiagnosed conditions, poorly understood by healthcare professionals, which often leads to long delays in diagnosis or misdiagnosis and to obstacles in the development of specific therapies.

The knowledge we currently have about the natural history, burden of the disease and mortality of these patients is based fundamentally on studies limited to small samples or studies focused on a specific lipodystrophy subtype (5–12). To the best of our knowledge, only one study, focused on five specific centres across three countries (Turkey, Brazil and United States) included patients with both generalised and partial lipodystrophy with a larger sample size (13).

In light of the previously-mentioned knowledge gap, the aim of this study was to assess the natural course and comorbidities of patients with generalised and partial lipodystrophies in Spain (both acquired and congenital) in an effort to contribute to the understanding of these syndromes.

## 2 Materials and methods

### 2.1 Study population

Of a total of 343 patients with rare adipose tissue disorders, 140 subjects were diagnosed with lipodystrophy and evaluated between 2001 and 2020 in the leading management and treatment centre for lipodystrophies in Spain (UETeM, CiMUS), which receives patients from the whole country.

**Figure 1:**
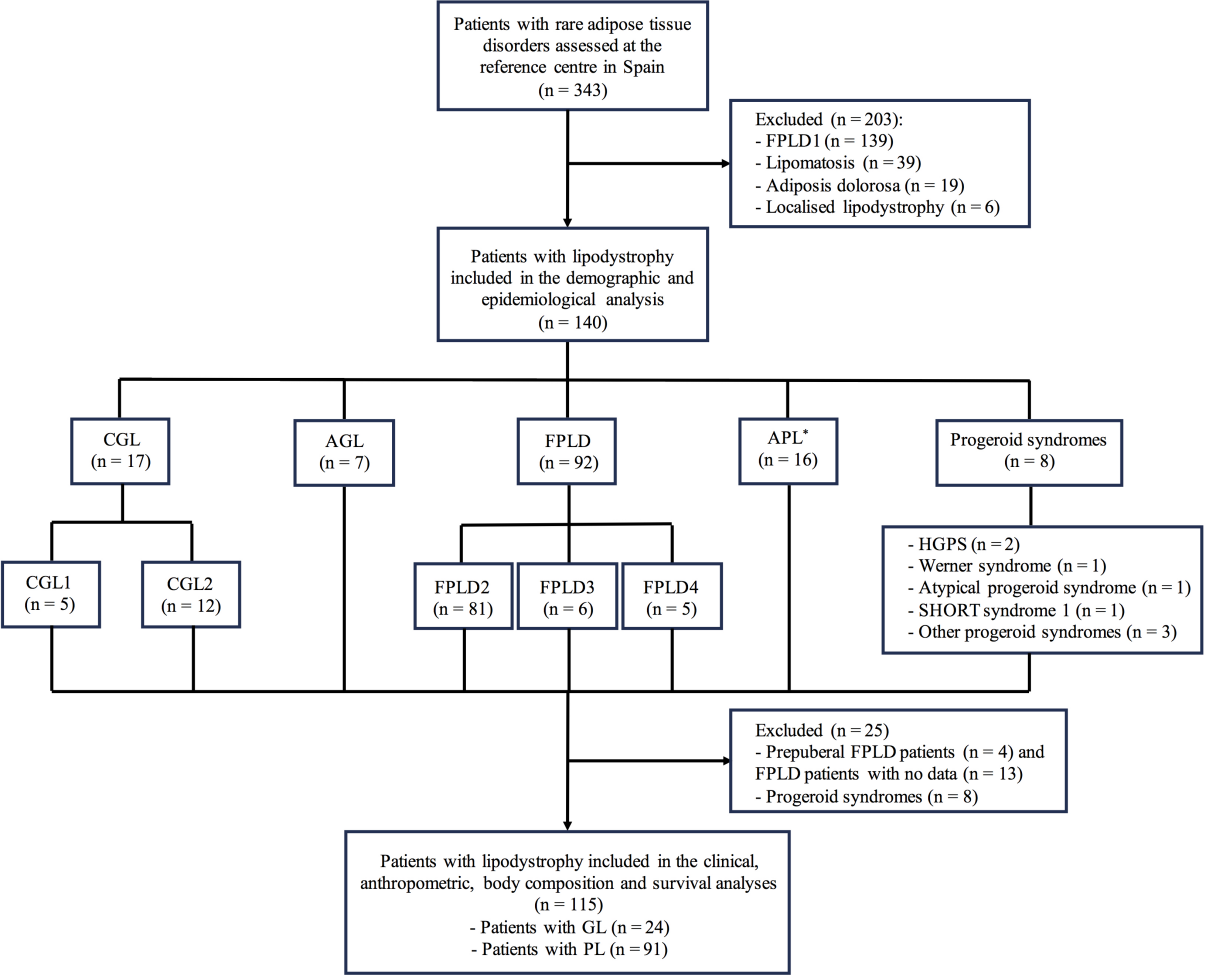
Flow diagram of patients with lipodystrophy evaluated at the reference centre in Spain. ^*^The group of patients with acquired partial lipodystrophy included 15 subjects with Barraquer-Simons syndrome and one subject with partial lipodystrophy after bone marrow transplantation and radiotherapy. CGL, congenital generalised lipodystrophy; CGL1, congenital generalised lipodystrophy type 1; CGL2, congenital generalised lipodystrophy type 2; FPLD, familial partial lipodystrophy; FPLD1, familial partial lipodystrophy type 1; FPLD2, familial partial lipodystrophy type 2; FPLD3, familial partial lipodystrophy type 3; FPLD4, familial partial lipodystrophy type 4; AGL, acquired generalised lipodystrophy; APL, acquired partial lipodystrophy; HGPS, Hutchinson-Gilford Progeria Syndrome; SHORT, short stature, hyperextensibility, ocular depression, Rieger anomaly, and teething delay; GL, generalised lipodystrophy; PL, partial lipodystrophy.

Patients with localised lipodystrophy, lipomatosis and adiposis dolorosa were excluded from this analysis. Patients with HIV-related lipodystrophy are not evaluated at said centre and they were also not included in the analysis. Among patients with genetic lipodystrophy, subjects with familial partial lipodystrophy (FPLD) type 1 were also excluded taking into account the following reasons: its genetic origin is unknown and, therefore, its diagnosis is merely clinical; this specific FPLD subtype may be confused with android obesity in general practice and, therefore, it is rare for these patients to be referred for evaluation (80.6% of them come from the same geographic region in which the centre is located); the high number of patients with a diagnosis of FPLD type 1 would distort the prevalence results of the current study; most of the data on these patients have already been analysed and reported by our group (14). In addition, patients with progeroid syndromes associated with lipodystrophy and patients with a diagnosis of lipodystrophy but no signs of the disease taking into account their young age were only included for the demographic and epidemiological analysis, considering that their inclusion may also affect the prevalence results. More detailed data of the inclusion/exclusion of patients and the population evaluated can be seen in the flow diagram of **Figure 1**.

On the other hand, it should be noted that twelve patients included in the analysis (11 with generalised lipodystrophy and one with partial lipodystrophy) started treatment with recombinant human leptin during follow-up.

### 2.2 Study design

This is a retrospective, observational, longitudinal study, carried out in a single centre attending patients with lipodystrophy from all over Spain. All the analyses were executed for the overall sample, as well as for generalised and partial lipodystrophy cohorts. Clinical data were recorded for analysis from birth until loss of follow-up, death or closure of the database.

This study was approved by the ethics review panel of the Red Gallega de Comités de Ética de la Investigación (approval code 2017/477) and carried out according to the ethical guidelines of the Helsinki Declaration.

### 2.3 Diagnosis of lipodystrophy and clinical data collection

The loss of adipose tissue and the affected regions were clinically confirmed by the same expert evaluator in all cases. Clinical diagnosis of lipodystrophy was also supported by body composition imaging. The pattern of partial or total fat loss and its onset was taken into consideration for each of the lipodystrophy subtypes. The absence of family history was also taken into account in the acquired forms. Molecular analysis was carried out in all patients evaluated in the centre in order to confirm or discard a genetic origin of the disorder. Other causes associated with wasting or weight loss (such as cancer cachexia, malnutrition, malabsorption, anorexia nervosa, thyrotoxicosis or chronic infections) were ruled out.

Clinical data were obtained from the patients’ electronic medical records and were evaluated by the same two examiners following a standardised and homogeneous protocol. Of the patients evaluated, the proportion of subjects with more than three medical evaluations during follow-up was 62.1% in the overall sample (66.7% in the generalised lipodystrophy group and 50.4% in the partial lipodystrophy group).

Physical examination was performed on all participants in the study, establishing a routine in their evaluation to enable a homogeneous comparison between the lipodystrophy subgroups. Regarding comorbidities, data collection focused on capturing organ abnormalities mainly related with adipose tissue dysfunction, ectopic fat accumulation and insulin resistance characteristic of the disease. In addition, other global defects found in these patients, extending beyond the key organ systems, were also recorded. DM and gestational diabetes were defined according to the 2022 American Diabetes Association criteria (15). Foot examination for the diagnosis of diabetic peripheral neuropathy, starting at diagnosis of diabetes and annually thereafter, included visual inspection and pedal pulses, as well as the 10-g monofilament exam, determination of vibration with a 128-Hz tuning fork, temperature and/or pinprick sensation. Symptoms of diabetic autonomic neuropathy were also assessed (16). Causes of neuropathy other than diabetes were discarded. Active screening of atherosclerotic vascular disease (ASCVD) and other cardiac abnormalities (including rhythm disturbances, hypertrophic cardiomyopathy and valvulopathies) was made only if symptoms or signs of any of these disorders were present and not on a routine basis in asymptomatic patients. Polycystic Ovary Syndrome (PCOS) was diagnosed based on the 2006 proposal of the Androgen Excess and PCOS Society: the presence of hyperandrogenism (hirsutism and/or hyperandrogenaemia) and ovulatory dysfunction (oligo-anovulation and/or polycystic ovarian disease assessed by ultrasound) (17). Hepatic steatosis was measured using high resolution ultra-sound B-mode imaging with a convex transducer (frequency of 3.5–5 MHz). For the diagnosis of intellectual disability, cognitive evaluation tests, such as the Wechsler Adult Intelligence Scale (WAIS-IV), were carried out. The diagnosis of anxiety-depressive disorder was made according to the Diagnostic and Statistical Manual of Mental Disorders, fifth edition (DSM-5) and the 10^th^ revision of the International Statistical Classification of Diseases and Related Health Problems (ICD-10) criteria (18). The ultimate diagnosis of the latter two conditions was made after referral to a psychiatry specialist in the centre. Progressive encephalopathy with or without lipodystrophy (PELD) was diagnosed in patients with pathogenic variants on chromosome 11q13 of the *BSCL2* gene and developmental regression of motor and cognitive skills in the first years of life, leading to severe progressive neurodegeneration and death in the first decade of life (19).

### 2.4 Anthropometry and body composition analysis

Height and weight were verified with digital scales and a stadiometer after 12 h overnight fasting and body mass index (BMI) was calculated. Waist and hip circumferences were determined using a soft tape measure by a single examiner. Waist circumference was calculated taking as a reference the superior border of the iliac crest, and hip circumference taking as a reference the greater trochanter. The patients’ skinfolds were measured using a Lange skinfold calliper (Cambridge Scientific Industries, Cambridge, MD, USA) in the same hemibody by a single examiner. The mean of three consecutive determinations was obtained.

Determination of fat mass and fat-free mass (FFM), both total and segmental, was performed using Dual-energy X-ray absorptiometry (DXA) with a Lunar DPX model (GE Healthcare Lunar, Madison, WI, USA) between 8:30-10:30 a.m. after 12 h of overnight fasting, avoiding previous excessive physical effort.

### 2.5 Analytical measurements

Laboratory results associated to metabolic complications were documented. Blood samples were taken between 8:00-9:00 a.m. after 12 h overnight fasting. Glucose, creatinine, creatinine kinase, triglycerides and total and fractionated cholesterol levels were measured by standardised methods with appropriate quality control and quality assurance procedures. Aspartate aminotransferase (AST), alanine aminotransferase (ALT) and γ-glutamyl transpeptidase (GGT) were determined with enzymatic methods on an ADVIA analyser (Siemens, Bayer Diagnostics, Tarrytown, NY, USA). Glycated haemoglobin (HbA1c) was measured with ion-exchange high-performance liquid chromatography (Bio-Rad Laboratories Inc., Hercules, CA, USA). Plasma insulin concentrations were determined in duplicate by chemiluminescence using a commercial kit (Nichols Institute, San Juan Capistrano, CA, USA). The homeostatic model assessment for insulin resistance (HOMA-IR) was calculated using the following formula: (fasting glucose [mg/dL]/fasting insulin [mIU/L])/405. Plasma leptin levels and C-peptide were determined by enzyme-linked immunosorbent assay (ELISA) (DRG International Inc., Springfield, NJ, USA).

### 2.6 Molecular analysis

The search for variants in 26 genes involved in the aetiology of congenital lipodystrophies was made in all the patients evaluated in the centre by next-generation sequencing (NGS) (Ion torrent System, Thermo Fisher Scientific, Waltham, MA, USA) of the entire coding region of the genes and the flanking intronic regions (*ADRA2A, AGPAT2, AKT2, BANF1, BLM, BSCL2, CAV1, CIDEC, ERCC6, ERCC8, FBN1, KCNJ6, LIPE, LMNA, MFN2, PCYT1A, PIK3R1, PLIN1, POLD1, POLR3A, PPARG, PSMB8, PTRF, SPRTN, WRN, ZMPSTE24*). Patients with benign variants were discarded using databases such as Varsome, ClinVar, gnomAD and dbSNP. In cases in which no results were obtained, SIFT and Polyphen2 were used. If no reported damaging/deleterious variants were found in the databases evaluated, the characteristic phenotype of the disease was taken into consideration. **Supplementary Table 1** lists the different variants in the genes that cause the disease in the patients with genetic lipodystrophy included in the study.

### 2.7 Statistical analysis

Data are expressed as the mean ± standard deviation (SD), median and interquartile range (IQR) or as n (%) values. For each continuous variable, the hypothesis of a normal distribution was verified by the Kolmogorov-Smirnov test. The χ^2^ test and Fisher’s exact test were used to compare qualitative variables in two groups. The t test or Mann-Whitney test were used to compare a quantitative variable in two groups. The analysis of covariance was also conducted to eliminate the possible modifying effect of certain covariates such as age and gender. Time to the onset of phenotype, time to the diagnosis of lipodystrophy, DM and hypertriglyceridaemia, and overall survival were described using a Kaplan-Meier curve. Log-rank tests were conducted to make the comparisons between patients with generalised and partial lipodystrophy. The level of significance was set at p < 0.05. All statistical analyses were performed with the SPSS 22.0 program (Chicago, IL, USA).

## 3 Results

### 3.1 Demographic and epidemiological data

A total of 140 patients were evaluated in the reference centre for lipodystrophies in Spain, with a median follow-up of 4.7 (0.5-17.6) years. Of these patients, 102 (72.9%) were women and 38 (27.1%) men (3:1 ratio). Age at first visit ranged from 5 months to 81.9 years of age.

A total of 117 (83.4%) patients presented genetic lipodystrophy and 23 (16.4%) acquired. According to the pedigree analysis, 62 index cases led to the detection of the rest of their affected relatives after cascade testing. As far as fat distribution is concerned, 108 (77.1%) patients presented partial lipodystrophy and 32 (22.9%) generalised lipodystrophy. As for the different lipodystrophy subtypes, 17 (12.1%) patients had congenital generalised lipodystrophy (CGL), 92 (65.7%) FPLD, 7 (5.0%) acquired generalised lipodystrophy (AGL), 16 (11.4%) acquired partial lipodystrophy (APL) and 8 (5.7%) patients had a progeroid syndrome. The most frequent disease was FPLD type 2 (57.9%), followed by Barraquer-Simons syndrome (10.7%) and CGL type 2 (8.6%). More detailed data on the distribution of the different lipodystrophy subtypes can be seen in **Figure 1**.

**Figure 2:**
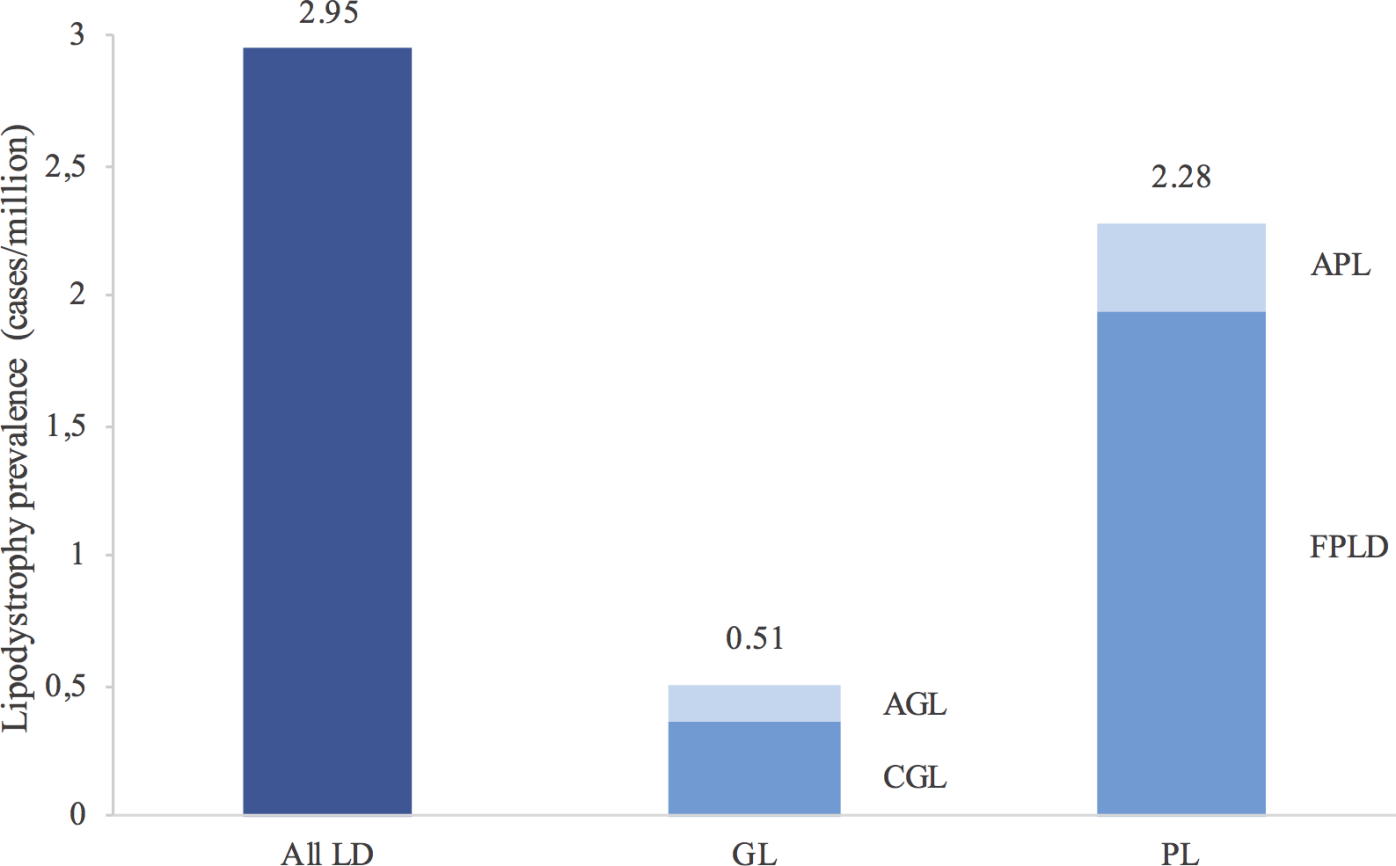
Estimated prevalence of lipodystrophies in Spain. Values above the bars report the prevalence. The estimated prevalence was calculated taking into account the population in Spain in 2020 according to the *Instituto Nacional de Estadística* (Spanish National Statistics Institute). Familial partial lipodystrophy type 1 has not been taken into account in the calculation of prevalence. LD, lipodystrophies; GL, generalised lipodystrophy; PL, partial lipodystrophy; AGL, acquired generalised lipodystrophy; CGL, congenital generalised lipodystrophy; APL, acquired partial lipodystrophy; FPLD, familial partial lipodystrophy.

The estimated prevalence of all lipodystrophy cases in Spain was 2.95 cases/million. When separated into generalised and partial lipodystrophy, the estimated prevalence was 0.51 and 2.28 cases/million, respectively (1.94 cases/million for FPLD, 0.34 cases/million for APL, 0.36 cases/million for CGL and 0.15 cases/million for AGL) (**Figure 2**).

In order to properly evaluate the natural course and comorbidities of lipodystrophies, subjects with progeroid syndrome, four prepuberal subjects with FPLD and 13 patients with FPLD and no data were discarded from the following analysis and, therefore, a total of 115 patients (91 with partial lipodystrophy and 24 with generalised lipodystrophy, 77.4% women, 45.2 ± 20.0 years of age) were subsequently analysed. The presence of women was predominant in the diagnosis of partial lipodystrophy (82.4%) in comparison with generalised lipodystrophy (58.3%) (p = 0.012), and patients with partial lipodystrophy were generally older (49.8 ± 18.4 vs 27.1 ± 15.5 years of age, p <0.0001), which is why the following non-demographic data were calculated after age and gender adjustment.

### 3.2 Clinical features

**Figure 3:**
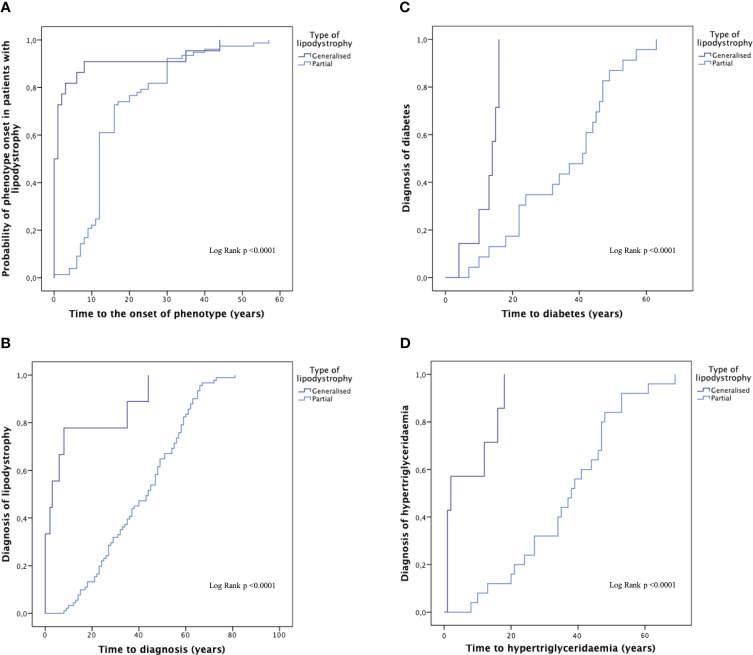
Time to the onset of phenotype, the diagnosis of the disease and the diagnosis of comorbidities in generalised and partial lipodystrophy. Time to the onset of phenotype **(A)**, time to diagnosis of the disease **(B)** and time to the diagnosis of diabetes mellitus **(C)** and hypertriglyceridaemia **(D)** in generalised and partial lipodystrophy. Subjects with progeroid syndrome, prepuberal subjects with familial partial lipodystrophy and patients with familial partial lipodystrophy and no data were excluded from this analysis.

The onset of phenotype occurred during childhood in 95.6% of patients with generalised lipodystrophy (4.7 ± 11.5 years of age; 47.8% at birth), and during adolescence-adulthood in 91.7% of patients with partial lipodystrophy (16.5 ± 10.8 years of age), p < 0.0001. The delay in diagnosis was 7.4 ± 8.1 years for patients with generalised lipodystrophy and 23.8 ± 17.1 years for patients with partial lipodystrophy (p < 0.0001) (**Figure 3**).

**Table 1** summarises the lipodystrophy-associated clinical features of the patients evaluated. Consanguinity was present in 4 out of 24 (16.7%) patients with generalised lipodystrophy and in only one case (1.1%) with partial lipodystrophy. The most frequent clinical features in the overall sample were phlebomegaly (70 patients; 60.9%), followed by muscle hypertrophy (62 patients; 53.9%) and then by signs of insulin resistance such as acanthosis nigricans (48 patients; 41.7%). Among patients with generalised lipodystrophy, phlebomegaly and umbilical protrusion/hernia were the most commonly reported clinical features (70.8% and 62.5%, respectively), whereas in the partial lipodystrophy cohort, phlebomegaly and muscle hypertrophy were the most frequent clinical characteristics reported (58.2% and 59.3%, respectively). Furthermore, there were several distinctive features of the generalised lipodystrophy group in comparison with the partial lipodystrophy group, such as acromegaloid features (7/24 patients [29.2%] with generalised lipodystrophy vs 4/91 patients [4.4%], p = 0.004), prognathism (4/24 patients [16.7%] with generalised lipodystrophy vs 2/91 patients [2.2%] with partial lipodystrophy, p = 0.043) and the presence of umbilical protrusion/hernia (15/24 patients [62.5%] with generalised lipodystrophy vs 4/91 [4.4%] patients with partial lipodystrophy, p <0.0001).

**Table 1:** Clinical features of patients with generalised and partial lipodystrophy.

	Overall (n=115)	Generalised lipodystrophy (n=24)	Partial lipodystrophy (n=91)	P value
Age (years)	45.2 ± 20.0	27.1 ± 15.5	49.8 ± 18.4	<0.0001^†^
Gender n (% women)	89 (77.4)	14 (58.3)	75 (82.4)	0.012^†^
Consanguinity n (%)	5 (4.3)	4 (16.7)	1 (1.1)	0.004^†^
Acromegaloid features n (%)	11 (9.6)	7 (29.2)	4 (4.4)	0.004^†^
Prognathism n (%)	6 (5.2)	4 (16.7)	2 (2.2)	0.043^†^
Ogival palate n (%)	5 (4.3)	3 (12.5)	2 (2.2)	0.171
Alopecia n (%)	7 (6.1)	0 (0.0)	7 (7.7)	0.538
Hirsutism n (%)	17 (14.8)	8 (33.3)	9 (9.9)	0.310
Hypoacusia n (%)	5 (4.3)	3 (12.5)	2 (2.2)	0.075
Acanthosis nigricans n (%)	48 (41.7)	13 (54.2)	35 (38.5)	0.978
Acrochordons n (%)	21 (18.3)	7 (29.2)	14 (15.4)	0.298
Hepatomegaly n (%)	42 (36.5)	12 (50.0)	30 (33.0)	0.075
Splenomegaly n (%)	9 (7.8)	5 (20.8)	4 (4.4)	0.056
Umbilical protrusion/hernia n (%)	19 (16.5)	15 (62.5)	4 (4.4)	<0.0001^†^
Phlebomegaly n (%)	70 (60.9)	17 (70.8)	53 (58.2)	0.213
Muscle hypertrophy n (%)	62 (53.9)	8 (33.3)	54 (59.3)	0.077
Myopathy n (%)	7 (6.1)	1 (4.2)	6 (6.6)	0.757
Muscle pain n (%)	25 (21.7)	4 (16.7)	21 (23.1)	0.799
Muscle contractures n (%)	6 (5.2)	1 (4.2)	5 (5.5)	0.999
Polyphagia n (%)	25 (21.7)	12 (50.0)	13 (14.3)	0.031^†^

Data are mean ± SD and n (%) values. Subjects with progeroid syndrome, prepuberal subjects with familial partial lipodystrophy and patients with familial partial lipodystrophy and no data were excluded from this analysis. Age and gender were used as covariables. ^†^p < 0.05.

### 3.3 Anthropometric measurements and body composition analysis

After age and gender adjustment, anthropometric parameters such as height, weight, waist and hip circumferences, as well as the skinfolds in the triceps, biceps, subscapular area, suprailiac area and thigh, were significantly lower in the generalised lipodystrophy cohort compared to the partial lipodystrophy cohort. However, no significant differences were found when measuring the skinfold of the calf in both groups. Regarding the DXA measurement of fat content, as expected, it was observed that, in patients with generalised lipodystrophy, this was significantly reduced both in total and in the limbs and trunk, compared to the partial lipodystrophy group. Patients with generalised lipodystrophy also presented higher FFM in the trunk, with no differences in the global analysis or in the upper and lower limbs (**Table 2**).

**Table 2:** Adjusted anthropometric and body composition data determined by Dual-energy X-ray absorptiometry (DXA) of lipodystrophy syndromes.

	Overall (n=115)	Generalised lipodystrophy (n=24)	Partial lipodystrophy (n=91)	P value
Height (cm)	157.7 ± 17.3	148.1 ± 31.4	159.8 ± 11.7	0.033^†^
Weight (kg)	63.3 ± 19.5	48.5 ± 21.9	66.5 ± 17.6	0.003^†^
BMI (kg/m^2^)	24.8 ± 6.3	20.7 ± 5.5	25.8 ± 6.1	0.008^†^
Waist perimeter (cm)	83.3 ± 16.0	70.9 ± 16.2	85.7 ± 14.9	0.004^†^
Hip perimeter (cm)	90.2 ± 13.3	79.5 ± 9.5	92.3 ± 13.0	0.003^†^
Triceps skinfold (mm)	8.9 ± 8.7	4.5 ± 2.9	9.9 ± 9.3	0.036^†^
Biceps skinfold (mm)	6.1 ± 5.2	2.8 ± 0.8	6.7 ± 5.4	0.012^†^
Subscapular skinfold (mm)	21.9 ± 15.7	7.7 ± 4.4	24.4 ± 15.5	0.001^†^
Suprailiac skinfold (mm)	13.6 ± 13.3	5.2 ± 1.9	15.3 ± 14.0	0.027^†^
Thigh skinfold (mm)	11.5 ± 13.9	5.7 ± 4.2	12.8 ± 14.9	0.033^†^
Calf skinfold (mm)	7.3 ± 8.3	4.2 ± 2.2	7.9 ± 9.0	0.051
Total fat (kg)	16.8 ± 10.8	4.4 ± 2.1	19.9 ± 9.8	<0.0001^†^
Total fat (%)	25.3 ± 11.8	9.9 ± 3.4	29.4 ± 9.6	<0.0001^†^
Upper-limb fat (kg)	1.8 ± 1.2	0.6 ± 0.4	2.1 ± 1.2	0.002^†^
Upper-limb fat (%)	24.4 ± 11.5	11.7 ± 6.7	27.8 ± 10.4	<0.0001^†^
Lower-limb fat (kg)	4.3 ± 3.6	1.4 ± 0.6	5.1 ± 3.6	0.003^†^
Lower-limb fat (%)	21.4 ± 11.9	10.9 ± 2.7	24.1 ± 11.9	<0.0001^†^
Trunk fat (kg)	9.9 ± 7.8	1.6 ± 1.1	12.1 ± 7.3	<0.0001^†^
Trunk fat (%)	26.8 ± 14.1	7.3 ± 4.2	31.9 ± 10.9	<0.0001^†^
Total FFM (kg)	42.4 ± 12.1	40.3 ± 17.8	42.9 ± 10.3	0.586
Upper-limb FFM (kg)	4.7 ± 1.8	4.4 ± 2.6	4.7 ± 1.5	0.634
Lower-limb FFM (kg)	13.0 ± 4.0	12.1 ± 6.0	13.3 ± 3.3	0.752
Trunk FFM (kg)	15.7 ± 7.8	21.0 ± 8.7	14.3 ± 7.8	<0.0001^†^

Data are mean ± SD values. Subjects with progeroid syndrome, prepuberal subjects with familial partial lipodystrophy and patients with familial partial lipodystrophy and no data were excluded from this analysis. Age and gender were used as covariables. ^†^p < 0.05. BMI, body mass index; FFM, fat-free mass.

### 3.4 Prevalence of main comorbidities

Data on the prevalence of lipodystrophy-associated comorbidities are shown in **Table 3**. Throughout the follow-up, DM was reported in 50 individuals (43.5%) in the overall sample, with no differences among the groups. Prevalence of DM complications was of 36.0% in the case of nephropathy (16.0% presenting renal failure and the rest of them isolated albuminuria, one patient with partial lipodystrophy required dialysis) and 20.0% in the case of retinopathy and peripheral neuropathy. Mean age at diagnosis of DM (**Figure 3**) was 30.2 ± 3.1 years in the overall sample (12.6 ± 1.6 years in patients with generalised lipodystrophy and 35.5 ± 3.2 years in patients with partial lipodystrophy; p <0.0001). As regards cardiovascular disease (**Table 3**), ischemic cardiopathy was present in 14 patients (15.4%) with partial lipodystrophy. Valvulopathy and hypertrophic cardiomyopathy were distinctive comorbidities associated to the generalised lipodystrophy group (eight patients [33.3%] and seven patients [29.2%], respectively) in comparison with the partial lipodystrophy cohort (p <0.0001). As far as neurological disease is concerned, intellectual disability was also more frequent among subjects with generalised lipodystrophy (11 patients [45.8%] vs one patient [1.1%] in the partial lipodystrophy group, p<0.0001). In addition, four (16.7%) patients with generalised lipodystrophy were diagnosed with PELD and, therefore, presented encephalopathy and gait disturbances. Hepatic steatosis was the most common liver abnormality identified, affecting 49 patients (42.6%) in the overall sample, with no differences among the groups. Episodes of acute pancreatitis were reported in three patients (12.5%) with generalised lipodystrophy and seven patients (7.7%) with partial lipodystrophy. As far as gynaecological disorders are concerned, 20 women (24.4%) manifested oligo/amenorrhea (four women [20.0%] in the generalised group and 16 [22.2%] in the partial lipodystrophy group) and 13 (15.9%) were diagnosed with PCOS. Anxiety-depressive disorder was diagnosed in 18 subjects (15.7%) of the overall sample, with 10 of them receiving chronic antidepressant and/or anxiolytic treatment. In all these cases patients related psychological distress due to their physical appearance as one of the causes. Other disorders detected with relative frequency in these patients are also shown in **Table 3**.

**Table 3:** Lifetime prevalence of organ abnormalities and comorbidities.

	Overall (n=115)	Generalised lipodystrophy (n=24)	Partial lipodystrophy (n=91)	P value
Diabetes mellitus and microvascular complications				
Gestational diabetes^*^ n (%)	10 (12.2)	2 (20.0)	8 (11.1)	0.327
DM n (%)	50 (43.5)	11 (45.8)	39 (42.9)	0.066
DM complications				
- Retinopathy n (%)	10 (20.0)	1 (9.1)	9 (23.1)	0.880
- Nephropathy n (%)	18 (36.0)	5 (45.5)	13 (33.3)	0.814
Renal failure n (%)	8 (16.0)	1 (9.1)	7 (17.9)	0.739
- Peripheral neuropathy n (%)	10 (20.0)	3 (27.3)	7 (17.9)	0.720
Cardiovascular disease				
Ischemic cardiopathy n (%)	14 (12.2)	0 (0.0)	14 (15.4)	0.495
Arrhythmia n (%)	4 (3.5)	1 (4.2)	3 (3.3)	0.793
Hypertrophic cardiomyopathy n (%)	7 (6.1)	7 (29.2)	0 (0.0)	<0.0001^†^
Valvulopathy n (%)	13 (11.3)	8 (33.3)	5 (5.5)	<0.0001^†^
Peripheral arterial disease n (%)	9 (7.8)	1 (4.2)	8 (8.8)	0.442
Neurological disease				
Encephalopathy n (%)	4 (3.5)	4 (16.7)	0 (0.0)	0.002^†^
Intellectual disability n (%)	12 (10.4)	11 (45.8)	1 (1.1)	<0.0001^†^
Gait disturbances n (%)	5 (4.3)	5 (20.8)	0 (0.0)	<0.0001^†^
Stroke n (%)	2 (1.7)	0 (0.0)	2 (2.2)	0.858
Abdominal abnormalities				
Hepatic steatosis n (%)	49 (42.6)	13 (54.2)	36 (39.6)	0.198
Cirrhosis n (%)	1 (0.9)	0 (0.0)	1 (1.1)	0.760
Cholelithiasis n (%)	6 (5.2)	0 (0.0)	6 (6.6)	0.503
Pancreatitis n (%)	10 (8.7)	3 (12.5)	7 (7.7)	0.492
Gynaecological disorders				
Oligo/amenorrhea^*^ n (%)	20 (24.4)	4 (40.0)	16 (22.2)	0.431
PCOS^*^ n (%)	13 (15.9)	1 (10.0)	12 (16.7)	0.319
Pregnancy loss^*^ n (%)	11 (13.4)	0 (0.0)	11 (15.3)	0.314
Uterine polyps/fibroids^*^ n (%)	9 (7.8)	1 (4.2)	8 (8.8)	0.815
Other disorders				
Hypertension n (%)	19 (16.5)	4 (16.7)	15 (16.5)	0.572
Anxiety-depressive disorder n (%)	18 (15.7)	1 (4.2)	17 (18.7)	0.418
Carpal tunnel Sd n (%)	8 (7.0)	1 (4.2)	7 (7.7)	0.617
Hypothyroidism n (%)	7 (6.1)	2 (8.3)	5 (5.5)	0.615
Goiter n (%)	13 (11.3)	1 (4.2)	12 (13.2)	0.530
Sleep apnoea n (%)	5 (4.3)	0 (0.0)	5 (5.5)	0.190
Malignancy n (%)	10 (8.7)	0 (0.0)	10 (11.0)	0.850

Data are n (%) values. Subjects with progeroid syndrome, prepuberal subjects with familial partial lipodystrophy and patients with familial partial lipodystrophy and no data were excluded from this analysis. Age and gender were used as covariables. ^†^p < 0.05. ^*^Among women. DM, diabetes mellitus; PCOS, polycystic ovary syndrome.

### 3.5 Analytical parameters

As for analytical measurements at first visit (**Table 4**), mean HbA1c among individuals with DM was 7.9 ± 2.2% in the overall sample, with HbA1c levels >7% occurring in around half of the patients (26 patients [52.0%]). Analytical parameters such as HOMA-IR showed results related with significant insulin resistance in these subjects. In terms of lipid profile, hypertriglyceridaemia was present both in patients with generalised and partial lipodystrophy. However, severe hypertriglyceridaemia was observed more frequently in the generalised lipodystrophy cohort, with triglyceride levels >500 mg/dl occurring in 7 out of 24 (29.2%) subjects with generalised lipodystrophy vs 5 out of 91 (5.5%) subjects with partial lipodystrophy, p = 0.011. In addition, mean age at diagnosis of hypertriglyceridaemia was 30.4 ± 3.3 in the overall sample (7.3 ± 2.9 years in patients with generalised lipodystrophy and 36.9 ± 3.1 years in patients with partial lipodystrophy; p <0.0001) (**Figure 3**). On the other hand, low-density lipoprotein cholesterol (LDL-C) and high-density lipoprotein cholesterol (HDL-C) levels were also significantly lower in the generalised group of subjects, with no differences in non-HDL-C or total cholesterol. As expected, lower leptin levels were observed in patients with generalised lipodystrophy in comparison with partial lipodystrophy. No differences were found regarding transaminase levels in both groups. The rest of the metabolic parameters analysed are shown in **Table 4**.

**Table 4:** Metabolic parameters of lipodystrophy syndromes at first visit.

	Overall (n=115)	Generalised lipodystrophy (n=24)	Partial lipodystrophy (n=91)	P value
Fasting glucose (mg/dL)	136.6 ± 83.7	136.5 ± 64.9	136.7 ± 89.5	0.557
HbA1c^*^ (%)	7.9 ± 2.2	8.6 ± 2.6	7.7 ± 2.1	0.444
- HbA1c > 7%^*^ n (%)	26 (52.0)	8 (72.7)	18 (46.1)	0.560
Insulin (mIU/L)	24.8 ± 24.8	38.8 ± 37.1	21.5 ± 19.8	0.067
HOMA-IR	9.6 ± 19.9	8.7 ± 7.9	9.9 ± 22.7	0.087
C-peptide (ng/mL)	2.8 ± 1.9	2.7 ± 1.9	2.8 ± 1.9	0.393
Total cholesterol (mg/dL)	180.9 ± 45.7	155.9 ± 46.3	188.7 ± 43.1	0.123
Non-HDL-C (mg/dL)	139.9 ± 45.5	123.0 ± 44.1	146.3 ± 44.9	0.290
LDL-C (mg/dL)	100.7 ± 38.5	71.8 ± 33.4	111.9 ± 34.5	0.002^†^
HDL-C (mg/dL)	40.4 ± 16.2	32.2 ± 15.5	43.7 ± 15.5	0.014^†^
Triglycerides (mg/dL)	152.0 (47.0-1937)	249.0 (55.0-1681.0)	142.5 (47.0-1937)	0.055
- Triglycerides >150 mg/dL n (%)	54 (46.9)	13 (54.2)	41 (45.0)	0.382
- Triglycerides >500 mg/dL n (%)	12 (10.4)	7 (29.2)	5 (5.5)	0.011^†^
GGT (IU/L)	19 (6.0-583.0)	21.0 (12.0-147.0)	19.0 (6.0-583.0)	0.869
AST (IU/L)	23.3 ± 13.9	27.4 ± 17.4	22.2 ± 12.9	0.674
ALT (IU/L)	20.0 (5.0-98.0)	32.5 (6.0-186.0)	23.5 (5.0-132.0)	0.158
Urea (mg/dL)	34.4 ± 15.5	29.7 ± 20.4	35.8 ± 13.7	0.888
Creatinine (mg/dL)	0.7 ± 0.7	0.5 ± 0.4	0.8 ± 0.7	0.724
CK (IU/L)	93 (34.0-916.0)	110.5 (45.0-916.0)	93.0 (34.0-331.0)	0.322
Leptin^**^ (μg/L)	0.3 (0.1-47.0)	0.3 (0.1-0.9)	4.3 (0.1-47.0)	<0.0001^†^

Data are mean ± SD or median (IQR) values and n (%) values. Subjects with progeroid syndrome, prepuberal subjects with familial partial lipodystrophy and patients with familial partial lipodystrophy and no data were excluded from this analysis. Age and gender were used as covariables. ^†^p < 0.05. ^*^Among patients with diabetes. ^**^Without or prior to leptin treatment. HbA1c, glycated haemoglobin; LDL-C, low-density lipoprotein cholesterol; HDL-C, high-density lipoprotein cholesterol; AST, aspartate aminotransferase; ALT, alanine aminotransferase; GGT, γ-glutamyl transpeptidase; CK, creatinine kinase.

### 3.6 Comparison between the two main subtypes of partial lipodystrophy

Taking into account the differences in the adipose tissue phenotype between the two main partial lipodystrophy subtypes (FPLD and APL), a subanalysis was made in order to compare body composition analysis and metabolic abnormalities (**Table 5**). Although there were no statistically significant differences in leptin levels or in the percentage of total fat, as expected, fat distribution differed between the two partial lipodystrophy subtypes. Thus, while the percentage of lower-limb fat was lower in the patients with FPLD, patients with APL showed lower trunk fat percentage. Consistent with this, subjects with FPLD also showed lower thigh and calf skinfolds and subjects with APL showed lower subscapular and suprailiac skinfolds (data not shown). A greater BMI was observed in the FPLD population, mainly based on greater FFM (both in total and in the limbs). The presence of phlebomegaly and muscle hypertrophy were also especially characteristic of the FPLD cohort (observed in 64.0% and 70.7% of these subjects, respectively). While DM was diagnosed in 37 (49.3%) patients with FPLD, it was only diagnosed in two (12.5%) patients with APL (p = 0.006), with worse metabolic control in the FPLD population (HbA1c 7.7 ± 2.2 vs 6.9 ± 0.0 in subjects with APL, p = 0.029). Although no statistically significant differences were found regarding organ abnormalities, peripheral arterial disease was only diagnosed in the FPLD cohort (10.7%) and a tendency towards greater triglyceride levels and towards a greater prevalence of hepatic steatosis was also observed for the FPLD subjects (44.0% vs 18.8% in comparison with patients with APL, p = 0.052). In addition, PCOS was likewise only diagnosed in women with FPLD (20.0%).

**Table 5:** Comparison of body composition and metabolic abnormalities between patients with familial partial lipodystrophy and acquired partial lipodystrophy.

	FPLD (n=75)	APL (n=16)	P value
Age (years)	51.3 ± 18.3	42.9 ± 18.0	0.108
Gender n (% women)	62 (82.7)	13 (81.3)	0.569
BMI (kg/m^2^)	26.8 ± 5.8	21.1 ± 5.3	0.001^†^
Total fat (%)	29.5 ± 9.8	29.1 ± 9.4	0.912
Upper-limb fat (%)	28.3 ± 9.4	26.1 ± 12.0	0.555
Lower-limb fat (%)	20.1 ± 9.4	36.6 ± 10.2	<0.0001^†^
Trunk fat (%)	34.4 ± 9.8	24.2 ± 10.6	0.006^†^
Total FFM (kg)	44.9 ± 9.2	34.4 ± 10.4	0.006^†^
Upper-limb FFM (kg)	5.0 ± 1.4	3.7 ± 1.5	0.020^†^
Lower-limb FFM (kg)	13.8 ± 3.0	11.4 ± 3.5	0.046^†^
Trunk FFM (kg)	13.5 ± 7.1	16.6 ± 5.2	0.178
Phlebomegaly n (%)	48 (64.0)	5 (31.3)	0.017^†^
Muscle hypertrophy n (%)	53 (70.7)	1 (6.3)	<0.0001^†^
DM n (%)	37 (49.3)	2 (12.5)	0.006^†^
Hypertension n (%)	13 (17.3)	2 (12.5)	0.482
Ischemic cardiopathy n (%)	12 (16.0)	2 (12.5)	0.536
Peripheral arterial disease n (%)	8 (10.7)	0 (0.0)	0.199
Stroke n (%)	2 (2.7)	0 (0.0)	0.678
Hepatic steatosis n (%)	33 (44.0)	3 (18.8)	0.052
Pancreatitis n (%)	5 (6.7)	2 (12.5)	0.357
PCOS^*^ n (%)	12 (20.0)	0 (0.0)	0.091
HbA1c^**^ (%)	7.7 ± 2.2	6.9 ± 0.0	0.029^†^
Triglycerides (mg/dL)	157.0 (55.0-1937.0)	74.0 (47.0-392)	0.067
Leptin^***^ (μg/L)	4.4 (0.1-47.0)	2.2 (1.7-14.0)	0.229

Data are mean ± SD or median (IQR) values and n (%) values. Prepuberal subjects with familial partial lipodystrophy and patients with familial partial lipodystrophy and no data were excluded from this analysis. ^†^p < 0.05. ^*^Among women. ^**^Among patients with diabetes. ^***^Without or prior to leptin treatment. FPLD, familial partial lipodystrophy; APL, acquired partial lipodystrophy; BMI, body mass index; FFM, fat-free mass; DM, diabetes mellitus; PCOS, polycystic ovary syndrome; HbA1c, glycated haemoglobin.

### 3.7 Overall survival

**Figure 4:**
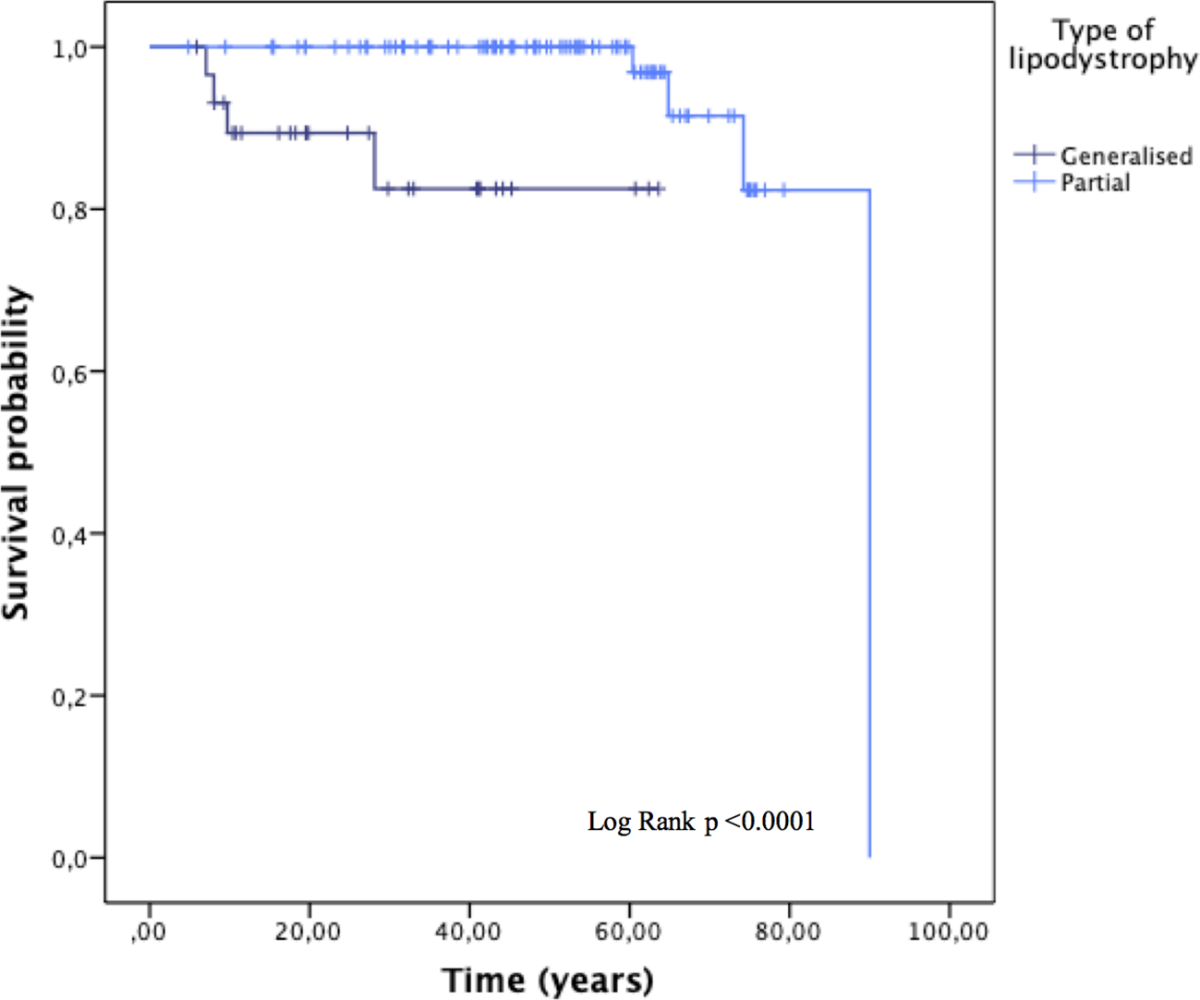
Overall survival, stratified by the type of lipodystrophy (generalised or partial). Subjects with progeroid syndrome, 13 patients with familial partial lipodystrophy and no data and four prepuberal subjects with familial partial lipodystrophy according to molecular analysis in whom the disease phenotype has not yet been developed, were excluded from this analysis.

During the follow-up, there were a total of eight deaths (6.8%); four (3.4%) in patients with generalised lipodystrophy and four (3.4%) in patients with partial lipodystrophy. Mortality status was unknown for 15 patients. Overall mean time to death was 83.8 ± 2.5 years (**Figure 4**). Furthermore, mean time to death was shorter among patients with generalised lipodystrophy in comparison to patients with partial lipodystrophy (55.3 ± 3.8 vs 86.2 ± 2.3 years, respectively; p <0.0001).

The causes of death of the patients with generalised lipodystrophy (CGL and AGL) were fundamentally respiratory tract infections. The three patients with pathogenic variants in the *BSCL2* gene who died presented PELD and died in the context of neurodegeneration, the deterioration of their general state due to respiratory sepsis or due to a status epilepticus not reversible with anticonvulsants. Among the patients with partial lipodystrophy, two of them died due to colorectal and gastric cancer and the other two due to myocardial infarction. More details are shown in **Table 6**.

**Table 6:** Causes of mortality of lipodystrophy patients.

Patient number	Age at death (years)	Gender	Subtype of lipodystrophy	Cause of death
1	8	Female	CGL2	Epileptic crisis, bronchial aspiration
2	10	Female	CGL2	Epileptic crisis
3	7	Male	CGL2	Respiratory infection
4	28	Male	AGL	Respiratory infection
5	64	Male	FPLD2	Myocardial infarction
6	74	Female	FPLD2	Myocardial infarction
7	90	Female	FPLD2	Colorectal cancer
8	61	Male	FPLD2	Gastric cancer

CGL2, congenital generalised lipodystrophy type 2; AGL, acquired generalised lipodystrophy; FPLD2, familial partial lipodystrophy type 2.

## 4 Discussion

The current study offers a comprehensive report of the status of lipodystrophy syndromes in Spain and adds more information about their natural course and comorbidities depending on fat distribution in order to contribute to increasing knowledge of these ultra-rare disorders.

In the current analysis, the estimated prevalence of lipodystrophies in Spain, according to the patients referred to its reference centre, was considered to be 2.95 cases/million (0.51 cases/million for generalised lipodystrophy [AGL and CGL] and 2.28 cases/million for partial lipodystrophy [APL and FPLD]). This is in line with a previous study in which, by evaluating the prevalence of lipodystrophy syndromes from five EMR database searches and four literature searches (also excluding HIV-related lipodystrophies), a range of prevalence for all lipodystrophies of 1.3–4.7 cases/million (for generalised lipodystrophy of 0.2–1.0 cases/million, and for partial lipodystrophy of 1.7–2.8 cases/million) was estimated (3). In a more recent analysis, using not only clinical but also molecular searches in the Geisinger Health System and MarketScan databases, a much higher prevalence for lipodystrophy syndromes was reported (47.3 cases/million in global and 1:7,588 for autosomal dominant FPLD) (4). One limitation of these database search analyses (in addition to probably including patients with FPLD type 1 without a firm diagnosis) is the possibility that cases of localised lipoatrophy or insulin lipoatrophy were also captured, which may likewise contribute to an increase in these estimated prevalences. There is definitely great difficulty in making a realistic estimate of a group of rare and heterogeneous diseases, and only large and prolonged registries will make it possible to present a more objective picture of the true prevalence.

Initial clinical characteristics were typically identified during childhood for patients with generalised lipodystrophy and during adolescence-adulthood for patients with partial lipodystrophy. In a systematic review, lower mean ages of onset for lipodystrophy have been reported. However, these results may not be directly comparable as the study population was limited to patients ≤ 18 years of age (20). The delay in the diagnosis in our sample was considerable for both groups of patients, but especially for subjects with partial lipodystrophy, taking into account that their particular phenotype may sometimes be subtle, making the diagnosis of the disease challenging (21), and probably due to the lack of recognition and understanding of these syndromes.

The most frequent clinical characteristics in the overall sample were phlebomegaly, muscle hypertrophy and acanthosis nigricans, common features in most lipodystrophy syndromes (1, 21–23). In addition, acromegaloid features, prognathism and the presence of umbilical protrusion/hernia were distinctive characteristics of patients with generalised loss of fat, as previously described in the literature (1, 24). Therefore, these specific clinical findings should be highlighted as important and useful features to take into account when making the clinical and differential diagnosis of these disorders. In addition, these lipodystrophy-associated changes in physical appearance can cause general physical discomfort, substantial psychological distress and, in consequence, have a negative impact on quality of life (25–27). In fact, anxiety-depressive disorder was diagnosed in 15.7% of our overall sample.

Beyond a physical examination, skinfold thickness measurement and DXA can provide information on the pattern of fat loss. As expected, total fat was significantly reduced in patients with generalised lipodystrophy compared to the partial lipodystrophy group. However, although significant differences were also found regarding fat loss in the lower limbs according to DXA (lower in the group of generalised lipodystrophy), skinfold thickness measurement showed no differences regarding calf skinfold in both groups. This could be due to the fact that DXA does not stratify the limbs by segments, but rather assesses the proximal and distal parts of lower limbs as a whole.

On the other hand, patients with generalised lipodystrophy developed their first metabolic abnormalities, such as DM, sooner than subjects with partial lipodystrophy. However, DM was finally reported in almost half of our sample with no differences among the groups. Dyslipidaemia in patients with lipodystrophy is characterised by hypertriglyceridaemia and low levels of HDL-C (28). Although high triglyceride levels were characteristic of both generalised and partial lipodystrophy in our study, severe hypetriglyceridaemia (triglycerides >500 mg/dl) was observed more frequently in the generalised lipodystrophy group and, in addition, it appeared earlier in these subjects, supporting the view that patients with generalised forms of lipodystrophy may have a greater severity in their metabolic complications (1, 5, 26). However, the manifestation of lipodystrophy syndromes is heterogeneous, and cases of partial lipodystrophy with comparable or more severe symptomology have also been documented (29, 30). In addition, not only HDL-C but also LDL-C levels were likewise significantly lower in patients with generalised lipodystrophy in our sample, with no differences in non-HDL-C or total cholesterol. This may suggest that other lipoproteins that are part of non-HDL-C, unlike LDL-C, are likely to be elevated in these patients and the fact that subjects with partial lipodystrophy had higher LDL-C levels could be due to increased intra-abdominal fat deposition. In relation with a greater severity of hypertriglyceridaemia in patients with generalised loss of fat, the prevalence of acute pancreatitis in this group of subjects was 12.5%, in comparison with 7.7% in partial lipodystrophy. Although no statistically significant differences were found, this was considerably higher than in the general population (31, 32) and in accordance with what has previously been reported in the literature, with an estimated prevalence of pancreatitis in generalised lipodystrophy of around 12% (6, 13).

Due to the increased prevalence of dyslipidaemia and DM, patients with lipodystrophy also appear to be at high risk of ASCVD (28). However, because these disorders are rare, only limited data from some cohorts of patients are available concerning the prevalence of atherosclerotic vascular complications (26). Thus, in our cohort, ischemic cardiopathy was present in 15.4% of cases with partial lipodystrophy and in none with generalised lipodystrophy. In this sense, although it is necessary to take into account the influence of the younger age of the generalised lipodystrophy group, a recent study conducted in 19 subjects with CGL types 1 and 2 quantifying coronary arterial calcification found that despite also evaluating a young CGL population, a quarter of them already presented an altered coronary calcium score, suggesting a potential increase in cardiovascular risk (33). On the other hand, while there are only a few anecdotal reports of ASCVD among CGL, AGL and APL patients, there is more substantial literature on ASCVD among patients with FPLD (26, 34, 35). This is consistent with the results observed in the current study. Thus, the prevalence of atherosclerotic cardiovascular events in these subjects ranged from 14% to 68% throughout the literature (12, 36–39), which, although variable, is also clearly higher than that reported for the general population (40). In addition, a higher frequency of cardiac events has likewise been observed in non-R482 FPLD type 2 carriers in comparison with FPLD type 2 subjects with variants at the 482nd codon (37, 41, 42). In this sense, in our cohort, 32/81 patients with FPLD type 2 harboured R482 pathogenic variants in the *LMNA* gene. On the contrary, while no differences were found regarding rhythm disturbances between both cohorts, hypertrophic cardiomyopathy and valvulopathy were distinctive comorbidities associated to the generalised lipodystrophy group in our sample, with a prevalence of 29.2% and 33.3%, respectively, which is higher than the previous estimates in the literature for these patients. In this sense, in a multicentre study evaluating a total of 230 subjects with these syndromes, cardiomyopathy was detected in 15.9% of patients with generalised lipodystrophy in comparison with 1.0% of patients with partial lipodystrophy (13). In another study focused on patients with CGL, cardiomyopathy was diagnosed in 2 out of 11 patients with CGL type 2 and in none with CGL type 1 or type 4 (6). Regarding liver abnormalities, hepatomegaly is commonly observed in patients with lipodystrophy (43). In a systematic review, it was shown that the rates of hepatomegaly are generally higher in generalised than in partial syndromes (7, 20, 44). However, no significant differences were found between both groups of patients in the present study. In addition, it has also been shown that the severity of metabolic liver disease (including hepatic steatosis) may depend on the type of lipodystrophy, being more severe in generalised lipodystrophy and presenting higher liver enzymes (44, 45). In our study, although hepatic steatosis was the most common liver abnormality identified, affecting 42.6% of the overall sample, its prevalence was lower than previously reported in other countries for both generalised and partial lipodystrophy (61.7%) (13). In addition, no differences were found between both groups of patients in our cohort. Neither were differences observed between generalised and partial lipodystrophy in another study in which the presence of hepatic steatosis was evaluated using magnetic resonance spectroscopy imaging, although in that study the possible influence of youth in the generalised lipodystrophy group on these results is emphasised (46), as is the case in our analysis. Another consideration that could explain the lack of differences between the generalised and partial lipodystrophy groups is the possible influence of the subjects with AGL, who usually have less severe hepatic steatosis than CGL patients (46). In addition, the metreleptin therapy initiated throughout follow-up in 11 patients with generalised lipodystrophy in our cohort should also be taken into consideration, as this treatment improves liver disease (25, 47). In fact, the previously-mentioned study reporting a greater overall prevalence of hepatic steatosis selected patients who have never received metreleptin therapy or other lipodystrophy-specific therapies, which could also explain these differences (13).

Although, as previously mentioned, generalised lipodystrophy is typically associated to a more severe metabolic disease in proportion with the extent of fat loss or dysfunction, it has been demonstrated that it also depends on the regions affected. Thus, while the adipose tissue of the lower body does not correlate with insulin resistance and may even protect against metabolic dysfunction (48), the lipid accumulation within the abdominal adipose tissue has been linked with insulin resistance and metabolic disease (49, 50). In other words, it could also be said that upper-body fat loss is less prone to be associated with metabolic disease than gluteofemoral fat loss. Thus, in the case of APL, unlike FPLD, as can be observed in the current analysis, comorbidities associated with insulin resistance are not usually a feature, unless fat loss extends down to the gluteofemoral adipose depots or when affected patients gain weight and become obese (7).

As for survival, the estimates of mean time to death in this population correspond, surprisingly, to the mean life expectancy for the general population in Spain according to the Spanish National Statistics Institute. However, it was clearly shorter for patients with generalised lipodystrophy, causing a loss of around 30 years of life in these individuals. This is in agreement with a previous study focused on patients with CGL, for whom a life expectancy of 62.9 ± 4.8 years was also calculated (10). Although lower estimates have been found for the generalised lipodystrophy cohort presented in the current analysis, the influence of the presence of PELD in three cases must be taken into consideration in these results, which led to an early death during middle childhood and could bias the results. On the other hand, in contrast with the overall mean time to death reported in the current study (83.8 ± 2.5 years), in another multicentre study analysing survival in a cohort of 230 patients with lipodystrophy from the USA, Turkey and Brazil, mean time to death was 63.9 ± 1.2 years (13). Severe insulin resistance and associated comorbidities (DM, dyslipidaemia, liver abnormalities, kidney disease and cardiovascular disease) are considered to be the major contributors to the severity of the disease (51). However, deaths due to chronic complications of DM and dyslipidaemia were only observed in two patients with FPLD type 2, deceased due to myocardial infarction. Among the factors that may have contributed to these differences in the mean life expectancy and in the lower prevalence of metabolic comorbidities as a cause of death, it could be speculated that the influence of a different lifestyle and the priority use of drugs with demonstrated reduction in cardiovascular events in recent years among different countries may be included. Furthermore, it should be taken into account that of the total number of patients finally evaluated in the comorbidities and survival analyses, 61 were diagnosed through cascade testing after the detection of the index cases with a clearly developed phenotype and, therefore, the metabolic abnormalities leading to death may not yet have developed or evolved in some of these subjects during the follow-up time. In addition, the young age of the patients with generalised lipodystrophy in our cohort may also influence the lack of time to develop end-organ complications and makes it difficult to estimate the actual mean time to death of these patients. In fact, the main cause of early death in patients with generalised lipodystrophy was respiratory infection, which is not surprising considering that three of these patients had PELD (52). However, in our cohort of patients, respiratory tract infection was also the cause of death of a 28-year-old patient with AGL with no neurological involvement of the disease. In fact, beyond neurodegeneration, deterioration of the general state and bronchoaspiration in patients with PELD, a high frequency of deaths due to infections (especially of respiratory origin) has been reported throughout the literature in generalised lipodystrophy, mainly in patients with CGL type 2 (10, 16, 53–55), which leads to the belief that there must be other potential mechanisms involved.

One limitation of this study could be the inclusion of 13 patients treated with metreleptin throughout the follow-up. However, excluding these subjects could also have created a selection bias in our sample population toward patients with less severe disease and, therefore, could have underestimated the prevalence rates of metabolic comorbidities. Nevertheless, the vast majority of the patients evaluated in this analysis are drug naïve, and the sample reflects real-world data. On the other hand, due to the reasons previously mentioned, the decision was taken to dispense with the FPLD type 1 group, which can contribute to varying the estimated prevalence of comorbidities in the partial lipodystrophy group. In addition, the inclusion of patients with FPLD type 1 would also increase the global prevalence for all lipodystrophies in Spain and make it even higher than previous estimates. Thus, there is a compelling need to better differentiate FPLD type 1, as there are no clear diagnostic criteria and confusion can be created regarding the prevalence of lipodystrophy syndromes in general.

In conclusion, the current longitudinal study shows the natural history and burden of the disease of both generalised and partial lipodystrophies in Spain, which may contribute towards augmenting knowledge of these rare syndromes, providing an initial point of comparison for upcoming results from ongoing prospective studies, such as the ECLip Registry study, among others (56, 57).

## Data availability statement

The original contributions presented in the study are included in the article/**Supplementary Material**, further inquiries can be directed to the corresponding author/s.

## Ethics statement

The studies involving humans were approved by Red Gallega de Comités de Ética de la Investigación. The studies were conducted in accordance with the local legislation and institutional requirements. Written informed consent for participation in this study was provided by the participants’ legal guardians/next of kin.

## Author contributions

AF-P and DA-V were responsible for the construction and the design of the study and for setting up the methodology. AC-P, LL and FC provided the necessary resources to conduct the study. AF-P, TP-M and ED-L were responsible for the data acquisition. AF-P and MG-V were responsible for the analysis of data and interpretation. AF-P was responsible for drafting the article. DA-V, SS-I, and SC-G were responsible for the review and editing of the manuscript. DA-V was responsible for the final approval of the version submitted. All authors contributed to the article and approved the submitted version.
