# Supplementary material for: Natural history and comorbidities of generalised and partial lipodystrophy syndromes in Spain
Source: Front Endocrinol (Lausanne). 2023 Nov 16;14:1250203. doi: 10.3389/fendo.2023.1250203 (PMC10687442; doi:10.3389/fendo.2023.1250203)
Supplement: Supplementary file 1 [file Table_1.docx]

Supplementary Material

Natural history and comorbidities of generalised and partial lipodystrophy syndromes in Spain

Antía Fernández-Pombo^1,2^, Sofía Sánchez-Iglesias^1^, Ana I. Castro-Pais^2,3^, Maria José Ginzo-Villamayor^4^, Silvia Cobelo-Gómez^1^, Teresa Prado-Moraña^1,2^, Everardo Josué Díaz-López^1,2^, Felipe F. Casanueva^3^, Lourdes Loidi^5^ and David Araújo-Vilar^1,2,*^

^1^UETeM-Molecular Pathology Group. Department of Psychiatry, Radiology, Public Health, Nursing and Medicine, IDIS-CiMUS, University of Santiago de Compostela, Spain.

^2^Division of Endocrinology and Nutrition. University Clinical Hospital of Santiago de Compostela, Spain.

^3^CIBER Fisiopatología de la Obesidad y la Nutrición (CIBERobn), 28029 Madrid, Spain

^4^Department of Estatística, Análise Matemática e Optimización, University of Santiago de Compostela, Spain.

^5^Galician Public Foundation for Genomic Medicine (SERGAS-Xunta de Galicia), Santiago de Compostela, Spain

*** Correspondence:**
David Araújo-Vilar, MD PhD
david.araujo@usc.es

**Table S1. Disease-causing variants detected in the genes associated with genetic lipodystrophy in the overall study population.**

| **Gene** | **cDNA** | **Protein** | **Phenotype** | **ACMG classification** |
| --- | --- | --- | --- | --- |
| *AGPAT2* | c.589-2A>G | p.(?)† | CGL1 | Pathogenic |
| *AGPAT2* | c.755_763del | p.(Met252_Thr254del) | CGL1 | Variant of uncertain significance |
| *AGPAT2* | c.317_588del | p.(Leu107Alafs*279) | CGL1 | Likely pathogenic |
| *AGPAT2* | c.158del | p.(Gly53Alafs*8) | CGL1 | Likely pathogenic |
| *BSCL2* | c.517dupA | p.(Thr173AsnfsTer5) | CGL2 | Pathogenic |
|  | c.385_386delinsGGA | p.(Pro129GlyfsTer11) |  | Likely pathogenic |
| *BSCL2* | c.517dupA | p.(Thr173AsnfsTer5) | CGL2 | Pathogenic |
| *BSCL2* | c.509_513del | p.(Tyr170Cysfs*6) | CGL2 | Pathogenic |
|  | c.985C>T | p.(Arg329Ter) |  | Pathogenic |
| *BSCL2* | c.517dupA | p.(Thr173AsnfsTer5) | CGL2 | Pathogenic |
|  | c.604C>T | p.(Arg202Ter) |  | Pathogenic |
| *BSCL2* | c.985C>T | p.(Arg329Ter) | CGL2 | Pathogenic |
|  | c.517dupA | p.(Thr173AsnfsTer5) |  | Pathogenic |
| *BSCL2* | c.985C>T | p.(Arg329Ter) | CGL2 | Pathogenic |
| *BSCL2* | c.974dup | p.(Ile326HisfsTer12) | CGL2 | Pathogenic |
| *LMNA* | c.1444C>T | p.(Arg482Trp) | FPLD2 | Pathogenic |
| *LMNA* | c.1396A>G | p.(Asn466Asp) | FPLD2 | Pathogenic |
| *LMNA* | c.1583C>T | p.(Thr528Met) | FPLD2 | Likely pathogenic |
| *LMNA* | c.1930C>T | p.(Arg644Cys) | FPLD2 | Likely pathogenic |
| *LMNA* | c.895A>G | p.(Ile299Val) | FPLD2 | Pathogenic |
| *LMNA* | c.1634G>A | p.(Arg545His) | FPLD2 | Likely pathogenic |
| *LMNA* | c.1445G>A | p.(Arg482Gln) | FPLD2 | Pathogenic |
| *LMNA* | c.1772G>T | p.(Cys591Phe) | FPLD2 | Variant of uncertain significance |
| *LMNA* | c.1583C>T | p.(Thr528Met) | FPLD2 | Likely pathogenic |
|  | c.1718C>T | p.(Ser573Leu) |  | Variant of uncertain significance |
| *LMNA* | c.1718C>T | p.(Ser573Leu) | FPLD2 | Variant of uncertain significance |
| *LMNA* | c.1804G>A | p.(Gly602Ser) | FPLD2 | Likely benign |
| *LMNA* | c.1458G>T | p.(Lys486Asn) | FPLD2 | Likely pathogenic |
| *LMNA* | c.82C>G | p.(Arg28Gly) | FPLD2 | Likely pathogenic |
| *LMNA* | c.1822G>A | p.(Gly608Ser) | HGPS | Pathogenic |
| *LMNA* | c.1824C>T | p.(Gly608=) | HGPS | Pathogenic |
| *LMNA* | c.29C>T | p.(Thr10Ile) | Atypical progeroid syndrome | Pathogenic |
| *PPARG* | c.335G>A | p.(Arg140His) | FPLD3 | Pathogenic |
| *PPARG* | c.663G>A | p.(Arg181Gln) | FPLD3 | Likely pathogenic |
| *PPARG* | c.1146C>A | p.(Ser410Arg) | FPLD3 | Pathogenic |
| *PPARG* | c.1400C>T | p.(Pro467Leu) | FPLD3 | Pathogenic |
| *PLIN1* | c.445G>A | p.(Gly149Arg) | FPLD4 | Variant of uncertain significance |
| *PLIN1* | c.1007C>T | p.(Ala366Val) | FPLD4 | Variant of uncertain significance |
| *PLIN1* | c.1168G>A | p.(Val390Ile) | FPLD4 | Variant of uncertain significance |
| *PLIN1* | c.1569A>G | p.(*523Trpext*15) | FPLD4 | Variant of uncertain significance |
| *RECQL2* | c.2886dup | p.(Ile963fs) | Werner syndrome | Likely pathogenic |
|  | c.1982-5del | Tyr660Ilefs7* |  | Benign |
| *RECQL2* | c.1161G>A | p.(Met387Ile) | Werner syndrome | Benign |
| *PIK3R1* | c.1945C>T | p.(R649W) | SHORT syndrome | Pathogenic |

Variants detected in the genes that cause the disease in the patients with genetic lipodystrophy included in the study. †: Intronic variant. CGL1: congenital generalised lipodystrophy; CGL2: congenital generalised lipodystrophy; FPLD2: familial partial lipodystrophy type 2; FPLD3: familial partial lipodystrophy type 3; FPLD4: familial partial lipodystrophy type 4; ACMG: American College of Medical Genetics.
